# Improving junior doctor medicine prescribing and patient safety: An intervention using personalised, structured, video‐enhanced feedback and deliberate practice

**DOI:** 10.1111/bcp.14325

**Published:** 2020-05-18

**Authors:** William Green, Muhammad Waseem Shahzad, Stephen Wood, Maria Martinez Martinez, Andrew Baines, Ahmad Navid, Robert Jay, Zara Whysall, John Sandars, Rakesh Patel

**Affiliations:** ^1^ University of Leicester School of Business University of Leicester Leicester UK; ^2^ Leicester General Hospital University Hospitals of Leicester (UHL) NHS Trust Leicester UK; ^3^ Pilgrim Hospital Boston United Lincolnshire Hospitals (ULH) NHS Trust Boston Lincolnshire UK; ^4^ Queen Elizabeth Hospital Birmingham University Hospitals Birmingham NHS Foundation Trust Edgbaston Birmingham UK; ^5^ School of Medicine University of Nottingham Nottingham UK; ^6^ Department of Human Resource Management, Nottingham Business School Nottingham Trent University Nottingham UK; ^7^ Health Research Institute, Faculty of Health, Social Care and Medicine Edge Hill University Ormskirk Lancashire UK

**Keywords:** avoidable harm, deliberate practice, foundation training, junior doctors, patient safety, prescribing, video‐enhanced feedback

## Abstract

**Aims:**

This research investigated the effectiveness of an intervention for improving the prescribing and patient safety behaviour among Foundation Year doctors. The intervention consisted of simulated clinical encounters with subsequent personalised, structured, video‐enhanced feedback and deliberate practice, undertaken at the start of four‐month sub‐specialty rotations.

**Methods:**

Three prospective, non‐randomised control intervention studies were conducted, within two secondary care NHS Trusts in England. The primary outcome measure, error rate per prescriber, was calculated using daily prescribing data. Prescribers were grouped to enable a comparison between experimental and control conditions using regression analysis. A break‐even analysis evaluated cost‐effectiveness.

**Results:**

There was no significant difference in error rates of novice prescribers who received the intervention when compared with those of experienced prescribers. Novice prescribers not participating in the intervention had significantly higher error rates (*P =* .026, 95% confidence interval [CI] Wald 0.093 to 1.436; *P =* .026, 95% CI 0.031 to 0.397) and patients seen by them experienced significantly higher prescribing error rates (*P =* .007, 95% CI 0.025 to 0.157). Conversely, patients seen by the novice prescribers who received the intervention experienced a significantly lower rate of significant errors compared to patients seen by the experienced prescribers (*P =* .04, 95% CI −0.068 to −0.001). The break‐even analysis demonstrates cost‐effectiveness for the intervention.

**Conclusion:**

Simulated clinical encounters using personalised, structured, video‐enhanced feedback and deliberate practice improves the prescribing and patient safety behaviour of Foundation Year doctors. The intervention is cost‐effective with potential to reduce avoidable harm.

**What is already known about this subject**

- Junior doctors in their Foundation Training are more likely to make prescribing errors than experienced healthcare professionals.
- Educational interventions are inconsistent at reducing prescribing errors among new prescribers such as Foundation Year doctors.
- The effectiveness of simulation‐based interventions for improving prescribing in practice is limited.

**What this study adds**

- Simulated clinical encounters with personalised, structured, video‐enhanced feedback using a deliberate practice approach support Foundation Year doctors to prescribe at a level consistent to experienced healthcare professionals.
- The intervention was implemented with consistent findings across different clinical sub‐specialty contexts in medicine (nephrology and renal transplantation) and surgery (general and orthopaedics).
- The intervention is cost effective and patients who were prescribed medication by Foundation Years who did not receive the intervention experienced significantly higher prescribing error rates.

## 1 INTRODUCTION

“Unsafe medication practices and medication errors are a leading cause of avoidable harm in health care systems across the world”.^1^ In response to the rise in the proportion of medicine‐related deaths and patient safety risks, the World Health Organisation (WHO) identified and announced *Medication Without Harm* as the third challenge faced by the World Alliance for Patient Safety in 2017.^2^ In England, medication errors due to adverse drug reactions are significant, consuming 181 626 bed days, causing 712 deaths and contributing to 1708 deaths in a year.^3^

As part of the effort to overcome medication‐related errors, one approach is to focus on prescribing. Prescribing is associated with medical errors, with high error rates across the world^4^, ^5^, ^6^, ^7^, ^8^ despite arguments of universal underreporting.^9^ Prescribing medication is an essential and complex skill^10^ for an increasingly wide range of prescribers including doctors, nurses and other prescribers. Prescribing involves the initiation, monitoring, continuation and modification of medication therapy, demanding a thorough understanding of clinical pharmacology as well as the judgement and ability to prescribe rationally for the benefit of patients.^11^ Against this backdrop, a prescribing error has been defined as “an unintentional significant (1) reduction in the probability of treatment being timely and effective or (2) an increase in the risk of harm when compared with generally accepted practice”. [^12^ p. 235]

Reducing avoidable harm from prescribing errors made by healthcare professionals should be a priority particularly among Foundation Year doctors (junior doctors in their first and second years of hospital training) who are more likely to make an error.^6^, ^13^ This group were found to make more prescribing errors than experienced colleagues,^6^ even accounting for the fact they prescribe more items in proportion to other grades and types of healthcare professionals. The Prescribing Safety Assessment was introduced in 2017 for medical students before graduating as a Foundation Year doctor,^14^ to minimise the contribution of knowledge deficits towards prescribing errors made in practice. Whilst interventions such as the Prescribing Safety Assessment^14^ ensure individuals have a minimum standard for some aspects related to prescribing, the longer‐term impact of the assessment on improving prescribing behaviour over time when individuals enter practice remains unknown.

The design and assessment of interventions for reducing prescribing errors among Foundation Year doctors needs to take into account the causal factors including the work environment (workload and time pressure), team factors (multiple individuals' involvement, communication, medicines reconciliation and documentation following incorrect instructions), task factors (poor availability of drug information at the time of admission, and support systems not available), individual factors (lack of personal knowledge and experience), and patient‐related factors (complexity of symptoms).^6^, ^15^, ^16^, ^17^, ^18^, ^19^, ^20^ This evidence also confirms that the causes of prescribing errors are multifactorial and multilevel, which is consistent with current understanding about human factors and error more generally across many safety‐critical industries.^21^ Previous systematic reviews evaluating the effectiveness of prescribing interventions have been inconclusive,^22^, ^23^, ^24^ probably because many of the studies appeared only to focus on a single target for interventions such as increasing prescribing knowledge. Consequently, the authors of these reviews have repeatedly called on further research to address the multiple factors identified from studies on human error, and to develop more sophisticated and multidimensional interventions that can address these contributing factors.^6^, ^15^, ^16^, ^17^, ^18^, ^19^, ^20^

Closer examination of some of the empirical studies included in these reviews identifies a number of methodological concerns. Only 19% of studies in one of the reviews distinguished between different grades of medical prescribers,^22^ therefore identifying what works for novice prescribers such as Foundation Year doctors remains unclear. Another review^23^ also confirmed that only 13% focused on new prescribers, meaning clear differences in the effectiveness of particular types or combinations of interventions cannot be deciphered for different grades or experience of prescriber. Many of these studies investigate the effectiveness of interventions in single prescribing contexts rather than across a range, limiting the transferability of the interventions. These single site studies typically involve pre‐ and post‐test interventions^23^ despite some reported limitations.^24^

### 1.1 Developing expertise through deliberate practice and feedback

Deliberate practice is an instructional approach for developing expertise where the goal is to develop mental models in the minds of learners for how they go about the planning, execution, monitoring and analysis of complex tasks such as prescribing.^25^, ^26^ There are examples of educational interventions underpinned by deliberate practice with particular effectiveness in terms of improved learning outcomes across a range of academic learning tasks^27^ and clinical skills.^28^, ^29^ A Best Evidence in Medical Education systematic review of high‐fidelity simulations spanning over three decades of research identified that learning outcomes that adopted deliberate practice were mixed.^29^ However, all were positive when feedback was provided as a structured activity alongside.^29^ The timing of feedback following performance on clinical simulations underpinned by deliberate practice is known to be important^30^ as well as the way in which it is given, that is, structured rather than informal or lacking a framework.^31^, ^32^

The way that feedback is delivered, that is, the medium through which it is being communicated is also important for improving potential outcomes.^33^ Video feedback has been demonstrated to have a positive performance effect across a wide range of areas—sport, music, communication and rehabilitation.^34^, ^35^, ^36^, ^37^ Within healthcare the use of video feedback has already been used in improving prescribing, medical and surgical outcomes.^38^, ^39^, ^40^, ^41^, ^42^, ^43^, ^44^ A study involving novices^45^ identified that video‐enhanced feedback improved clinical skills performance over and above the effect of receiving feedback on skills development directly from an expert. In another study involving a pharmacist‐led video‐stimulated feedback intervention, researchers also observed a reduction in prescribing errors among participants, albeit there was no control group to fully assess the real effect of the intervention.^46^

The aim of this research was to investigate the effectiveness of simulated clinical encounters using personalised, structured, video‐enhanced feedback and deliberate practice for improving the prescribing and patient safety behaviour of Foundation Year doctors.

## 2 METHODS

Our intervention (described in Appendix A) combined the simulation of a clinical encounter and the use of personalised, structured, video‐enhanced feedback. To facilitate deliberate practice, specific elements of practice for the doctors to focus on were identified through video viewing. The design of the simulated clinical encounters and simulation environment resembled an actual clinical environment in order to capture the authentic range of factors that contribute towards prescribing errors.^6^, ^14^, ^15^, ^16^, ^17^, ^18^, ^19^, ^20^, ^23^

### 2.1 Study sites

Three prospective, non‐randomised intervention studies were conducted across four‐month rotations in Nephrology and Renal Transplantation (Study 1), Surgery (Study 2) and Orthopaedics (Study 3) in two acute care NHS Trusts in England. Junior doctors on a Foundation and Core Training programme in the UK rotate through a different specialty every four months for two years. Experimental groups included *novice* junior doctors, defined as Foundation Year doctors; and those in their first and second year of training, on the East Midlands South Foundation Training and Core Medical Training programmes.^47^ Control groups comprised other prescribers (novices and experienced) working on the study wards but not participating in the intervention. Experienced prescribers comprised those who had completed their Foundation Training. The simulated clinical encounters were delivered over two days in the first week of a four‐month rotation. The personalised, structured, video‐enhanced feedback on prescribing behaviour was completed within the first month of the four‐month rotation.

### 2.2 Study design

A consistent approach to participant recruitment was adopted across the three study sites. Participants were invited to participate on a voluntary basis via email. The experimental groups were made up of cohorts rotating through the study sub‐specialty sites.

### 2.3 Study 1

This study had two objectives. First, to assess the value of personalised, structured, video‐enhanced feedback over and above any learning gained from participating in simulated clinical encounters alone. Second, to establish the cost‐effectiveness of the intervention on reducing avoidable harm. Two experimental groups and two control groups were constructed: Experimental Group 1—Foundation Year prescribers who participated in simulated clinical encounters; Experimental Group 2—Foundation Year prescribers who participated in simulated clinical encounters and received personalised, structured, video‐enhanced feedback; Novice Control—Foundation Year prescribers in their first and second year of practice; and Experienced Control—experienced prescribers beyond Foundation Years. To mitigate the knowledge‐sharing effect between the experimental groups, Experimental Group 1 participated in the rotation prior to Experimental Group 2.

Junior doctors rotating to the Department of Nephrology and Renal Transplantation, John Walls Renal Unit, Leicester General Hospital, Universities of Leicester NHS Trust, were invited to participate in the study. The department includes four inpatient wards with a total of 59 beds, with all patients admitted under specialist renal care. Simulated clinical encounters were conducted at the Robert Kilpatrick Clinical Sciences Building, Leicester Royal Infirmary, University Hospitals of Leicester NHS Trust.

### 2.4 Study 2

This study had one objective: to assess the prescribing behaviour of Foundation Year doctors following participation in the intervention at a second research site. The intervention delivered followed the treatment of Experimental Group 2 in Study 1: simulated clinical encounters with personalised, structured, video‐enhanced feedback. An Experimental Group of Foundation Year doctors who participated in simulated clinical encounters and received personalised, structured, video‐enhanced feedback was compared to an Experienced Control group of experienced prescribers beyond Foundation Years. Foundation Year doctors rotating to the Department of Surgery were invited to participate in the study. The department included 2 29‐bed inpatient wards. Simulated clinical encounters were conducted in an area of the Discharge Lounge not used for patient care at Pilgrim Hospital, United Lincolnshire Hospitals NHS Trust.

### 2.5 Study 3

This study had two objectives. First, to assess the prescribing behaviour of Foundation Year doctors following participation in the intervention at a third research site. The second objective sought to establish the impact of the participating doctors prescribing behaviour on patients, as one patient is likely to be prescribed items by multiple prescribers. Prescribing data were, therefore, analysed to compare prescribing error rates experienced by patients dependent on whether the individual items prescribed had been written by a prescriber belonging to a particular study group (denoted as *Patient‐level data* analysis).

To meet these two objectives, Study 3 repeated the intervention in the same hospital as Study 2 but in the Department of Orthopaedics. Junior doctors rotating to this department were invited to participate in the study. Data were compared across three groups: an Experimental Group of Foundation Year doctors who participated in simulated clinical encounters and received personalised, structured, video‐enhanced feedback; a Novice Control group of Foundation Year doctors; and an Experienced Control group of doctors beyond Foundation Year. The department included one elective surgery ward with 14 beds and one trauma ward with 28 beds.

### 2.6 Data collection

Medication orders across the three study sites were made on written prescriptions. Data were collected over a 4‐month rotation period for all prescribers. Pharmacists responsible for the inpatient wards participating in the studies collected data from inpatient drug charts. Data were collected as part of the pharmacists' daily activity and therefore prescribing errors were also corrected as part of their usual medicine reconciliation process. All prescribed medicine items reviewed by pharmacists were included in the data corpus. It is feasible, however, that some medicine items prescribed to patients were not reviewed, e.g. items prescribed to patients who were then transferred out of the study site, outside of the pharmacy data collection team working hours. All doctors working in the study sites over the duration of the research were aware prescribing was monitored by pharmacists as part of the usual medicines reconciliation process. Data collected were peer reviewed by the lead pharmacist at each study site. Anonymised patient‐level data were shared by the lead pharmacist with the study team for analysis.

A standardised prescribing error data collection form (Appendix B) was designed and piloted prior to Study 1 to ensure consistency across all sites.^12^ Discharge data were not included, similar to other research investigating written prescribing errors.^6^ The form required the pharmacy team to record the date a prescription was made, ward, patient details (initials and hospital number), prescriber details (initials, occupation and grade) and prescribing error details (drug name, dose and frequency, description of error, what, if any, doses were given, whether the error led to actual negative outcomes for the patient and the potential severity of error). The potential severity of error classification system used in the GMC‐funded EQUIP study,^6^ which was based on prior research,^48^, ^49^, ^50^, ^51^, ^52^ was also used for this research. This classification system uses four severities of error: minor, significant, serious and potentially lethal.

### 2.7 Data analysis

The independent variable in Study 1 measured group membership, differentiating between four groups: Experimental Group 1, Experimental Group 2, Novice Control Group and Experienced Control Group. The dependent variable was a count variable, number of errors per prescriber. The data were analysed using a negative binomial regression (to adjust for over dispersion of data) with the Experienced Control Group as the reference group. A break‐even analysis was conducted to establish the cost effectiveness of the intervention based on Study 1. The break‐even analysis is detailed in Appendix C.

The independent variable in Study 2 consisted of two groups: Experimental Group and Experienced Control Group. The dependent variable in Study 2 was the error rate per prescriber, calculated as the number of errors divided by total number of items prescribed by each individual prescriber. Therefore, these data accounted for the total number of items prescribed by all prescribers.

The independent variable in Study 3 consisted of three groups: Experimental Group, Novice Control Group and Experienced Control Group. The dependent variable in Study 3 was, as in Study 2, the error rate per prescriber, calculated as the number of errors divided by total number of items prescribed by each individual. Again, these data accounted for the total number of items prescribed by all prescribers.

Study 2 and 3 data were analysed using regression analysis in STATA 15 with the Experienced Control Group as the reference group. Given the experimental nature of the study design, these analyses enable causal links to be proposed^53^; however, causal links must be approached with caution due to the limitations of the nonrandomised study design.

Data were further analysed to establish the impact on patients of prescribing behaviours demonstrated by the three groups on the patients in Study 3. For this specific analysis, the dependent variable was error rate per patient, calculated as the number of errors (in items prescribed to each individual patient) divided by total number of items prescribed to each individual patient. The independent variable consisted of three groups (Experimental Group, Novice Control and Experienced Control) with the relevant number of patients seen by participants in each group. These data were analysed using regression analysis with the Experienced Control Group as the reference group.

### 2.8 Ethics

The study was undertaken and registered at the two NHS Trusts as part of their patient safety and improving quality clinical effectiveness programme, and Health Education England working across the East Midlands wider quality improvement and innovation initiative (study reference number LEI0085). The study did not require full NHS ethics approval.

### 2.9 Patient and public involvement

Patients affiliated to the Leicester Kidney Patient Association were invited to support Study 1. Patients were identified and invited by clinical leads to support Studies 2 and 3. Patients co‐designed and co‐delivered the simulated clinical encounters across all three study sites. Patients were involved in the local dissemination of outcomes from each study and the findings across all sites were presented by the lead author to the East Midlands Patient and Public Involvement Senate.

## 3 RESULTS

### 3.1 Study 1: Department of Nephrology and Renal Transplantation

#### 3.1.1 Participants

All junior doctors invited to the simulations participated: 11 without video‐enhanced feedback (Experimental Group 1: 7 FY1 and 4 FY2) and 13 with video‐enhanced feedback (Experimental Group 2: 8 FY1 and 5 FY2). Five participants were excluded from analysis as no prescribing data were attributable to them; 2 FY1 and 2 FY2 from Experimental Group 1 and 1 FY2 from Experimental Group 2 (see Table 1).

**TABLE 1:** Errors by group in Department of Nephrology and Renal Transplantation where Experimental Group 1 are Foundation Year doctors who participated in simulated clinical encounters; Experimental Group 2 are Foundation Year doctors who participated in simulated clinical encounters and received personalised, structured, video‐enhanced feedback; Novice Control are Foundation Year doctors; and Experienced Control are doctors beyond Foundation Training

	Experimental Group 1	Experimental Group 2	Novice Control	Experienced Control	Total
No. of prescribers	7	12	7	35	61
No. of total errors	102	72	67	156	397
Mean number of errors	14.57	6.00	9.57	4.46	6.51
Standard deviation of total errors	11.80	6.92	6.40	3.97	6.85
**Minor errors**					
No. of minor errors	41	32	25	67	165
Minor errors per prescriber	5.86	2.67	3.57	1.91	2.70
Standard deviation of minor errors	5.70	2.46	2.94	2.42	3.19
**Significant errors**					
No. of significant errors	52	34	21	74	181
Significant errors per prescriber	7.43	2.83	3.00	2.11	2.97
Standard deviation of significant errors	6.08	4.73	2.83	2.32	3.79
**Serious errors**					
No. of serious errors	9	6	20	15	50
Serious errors per prescriber	1.29	0.50	2.86	0.43	0.82
Standard deviation of serious errors	1.38	0.80	2.54	0.78	1.38
**Potentially lethal errors** *					
No. of potentially lethal errors	0	0	1	0	1
Potentially lethal errors per prescriber	0	0	0.14	0	0.02
Standard deviation of potentially lethal errors	0	0	0.38	0	0.13

* The observed lethal error in the Novice Control group was merged with the Novice Control group serious errors for the analysis.

#### 3.1.2 Prescribing data

**FIGURE 1:**
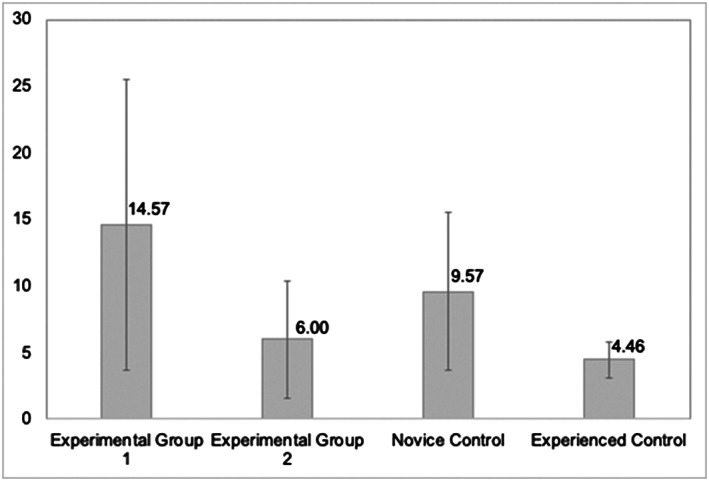
Errors per prescriber (y‐axis) with confidence intervals by group for Study 1: Department of Nephrology and Renal Transplantation

There was a significant difference in error rates between Experimental Group 1, the intervention group who did not receive video‐enhanced feedback, and Experienced Control (*P =* .006, 95% confidence interval [CI] Wald 3.36 to 2.034). Experimental Group 1 had the highest number of errors (14.57) per prescriber amongst all four groups (Table and Figure 1). However, there was no significant difference in error rates between Experimental Group 2, the intervention group who did receive the video‐enhanced feedback, and Experienced Control. There was a significantly higher number of errors per prescriber (9.57) among the Novice Control group compared to the Experienced Control group (*P =* .082, 95% CI Wald −0.096 to 1.625; Table and Figure 1).

Experimental Group 1 also made significantly more errors for both minor and significant error categories, compared with Experienced Control (*P =* .015, 95% CI Wald 1.24 to 7.52; and *P =* .005, 95% CI Wald 1.45 to 8.52 respectively). There was no significant difference in errors made between Experimental Group 2, Novice Control and Experienced Control across both categories of error type.

Novice Control had significantly higher serious errors per prescriber compared to Experienced Control (*P* < .001, 95% CI Wald 3.61 to 13.58). There was no significant difference in serious error rates between Experimental Group 2 and Experienced Control.

The break‐even analysis (Appendix C) calculated from the errors observed in the data demonstrates that the cost of the intervention is less than the potential cost attributed to the observed errors.

### 3.2 Study 2: Department of Surgery

#### 3.2.1 Participants

Fourteen junior doctors were invited and participated in the simulations and were included in the analysis (see Table 2).

**TABLE 2:** Errors by group in Department of Surgery where Experimental Group are Foundation Year doctors who participated in simulated clinical encounters and received personalised, structured, video‐enhanced feedback; and Experienced Control are doctors beyond Foundation Training

	Experimental Group	Experienced Control	Total
No. of prescribers	14	15	29
Items prescribed	7227	1371	8598
Items prescribed per prescriber	516.21	91.40	296.48
Standard deviation of items prescribed	144.75	123.21	252.97
**All categories of errors**			
Errors	915	223	1138
Errors per prescriber	65.36	14.87	39.24
Standard deviation of errors	26.59	26.72	36.67
Error rate per prescriber	13.07%	12.88%	12.97%
Standard deviation of error rate	4.64%	7.48%	6.16%
**Minor errors**			
No. of minor errors	723	172	895
Minor error rate per prescriber	10.58%	9.92%	10.24%
Standard deviation of minor error rate	4.09%	6.53%	5.41%
**Significant errors**			
No. of significant errors	191	51	242
Significant error rate per prescriber	3.14%	4.54%	3.86%
Standard deviation of significant error rate	1.40%	3.45%	2.71%
**Serious errors** *			
No. of serious errors	1	0	1
Serious error rate per prescriber	0.02%	0	0.01%
Standard deviation of serious error rate	0.01%	0	0.04%

* The observed serious error in the Experimental Group was merged with the Experimental Group significant errors for the analysis.

#### 3.2.2 Prescribing data

**FIGURE 2:**
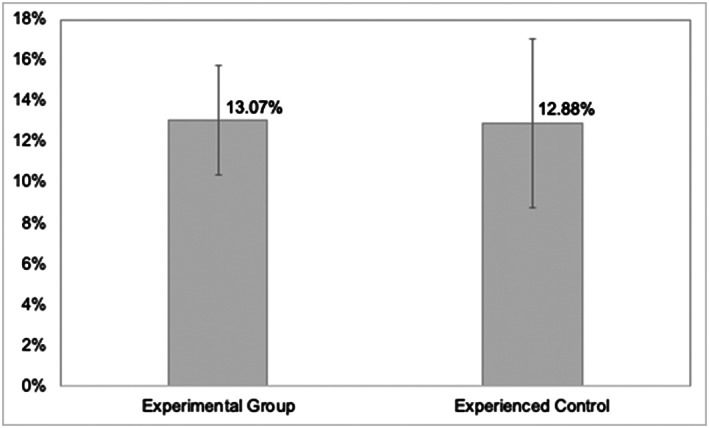
Error rates per prescriber (y‐axis) with confidence intervals by group for Study 2: Department of Surgery

There was no significant difference in error rates per prescriber between the Experimental Group comprising Foundation Year doctors and Experienced Control comprising experienced prescribers beyond Foundation Year (Table and Figure 2). This finding was observed despite a significantly higher level of prescribing activity for the Experimental Group (*p* < 0.001, 95% CI 322.63 to 526.99). There was no significant difference in error rates per prescriber across the various error severity types.

### 3.3 Study 3: Department of Orthopaedics

#### 3.3.1 Participants

The twelve junior doctors rotating through the Orthopaedic specialty were invited to participate. Seven were able to attend the simulations and constitute the Experimental Group. The remaining five could not participate due to clinical commitments and constitute the Novice Control (see Table 3).

**TABLE 3:** Errors by group in Department of Orthopaedics where Experimental Group are Foundation Year doctors who participated in simulated clinical encounters and received personalised, structured, video‐enhanced feedback; Novice Control are Foundation Year doctors; and Experienced Control are doctors beyond Foundation Training

	Experimental Group	Novice Control	Experienced Control	Total
No. of prescribers	7	5	3	15
Items prescribed	2203	1199	724	4126
Items prescribed per prescriber	314.71	239.80	241.33	275.07
Standard deviation of items prescribed	131.51	82.20	175.71	123.40
**All categories of errors**				
Errors	289	459	148	896
Errors per prescriber	41.29	91.80	49.33	59.73
Standard deviation of errors	12.9	31.2	45.4	34.7
Error rate per prescriber	16.14%	41.40%	20.00%	25.33%
Standard deviation of error rate	8.61%	16.49%	5.57%	15.95%
**Minor errors**				
No. of minor errors	245	418	119	782
Minor error rate per prescriber	14.21%	39.18%	16.49%	22.99%
Standard deviation of minor error rate	8.98%	16.77%	3.58%	16.06%
**Significant errors**				
No. of significant errors	43	40	28	111
Significant error rate per prescriber	2.46%	5.82%	5.18%	4.12%
Standard deviation of significant error rate	1.61%	5.70%	4.14%	3.94%
**Serious errors** *				
No. of serious errors	1	1	1	3
Serious error rate per prescriber	0.04%	0.25%	0.11%	0.12%
Standard deviation of serious error rate	0.10%	0.57%	0.20%	0.33%

* The observed serious errors were merged with the significant errors for the analysis.

**TABLE 4:** Errors for individual patients to determine differences between the group of doctors by whom the patients were seen/prescribed; Experimental Group are Foundation Year doctors who participated in simulated clinical encounters and received personalised, structured, video‐enhanced feedback; Novice Control are doctors in their first and second Foundation Year; and Experienced Control are doctors beyond Foundation Year

	Patients seen by Experimental Group	Patients seen by Novice Control	Patients seen by Experienced Control
Number of patients	265	178	111
Items prescribed	2203	1199	724
Items prescribed per patient	8.32	6.74	6.52
Standard deviation of items prescribed	6.81	5.85	5.63
**All categories of errors**			
Errors	273	419	137
Errors per patient	1.03	2.35	1.23
Standard deviation of errors	1.39	3.73	1.94
Error rate per patient	14.01%	28.38%	19.29%
Standard deviation of error rate	21.94%	34.61%	28.21%
**Minor errors** No. of minor errors	229	387	109
Minor errors per patient	0.86	2.17	0.98
Standard deviation of minor errors	1.17	3.60	1.53
Minor error rate per patient	12.42%	26.51%	16.67%
Standard deviation of minor error rate	20.77%	34.18%	26.49%
**Significant errors**			
No. of significant errors	43	31	27
Significant errors per patient	0.16	0.17	0.24
Standard deviation of significant errors	0.47	0.51	0.62
Significant error rate per patient	2.40%	5.28%	5.82%
Standard deviation of significant error rate	10.28%	18.38%	18.29%
**Serious errors** *			
No. of serious errors	1	1	1
Serious errors per patient	0.004	0.01	0.01
Standard deviation of serious errors	0.06	0.07	0.09
Serious error rate per patient	0.04%	0.19%	0.09%
Standard deviation of serious error rate	0.68%	2.50%	0.95%

* The observed serious errors were merged with the significant errors for the analysis.

#### 3.3.2 Prescribing data

**FIGURE 3:**
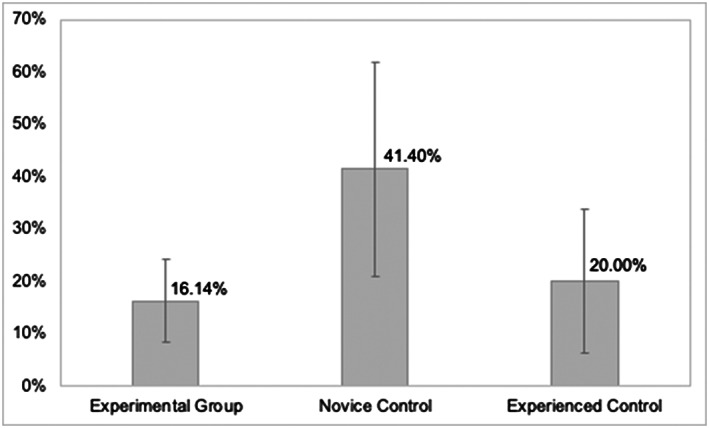
Error rates per prescriber (y‐axis) with confidence intervals by group for Study 3: Department of Orthopaedics

There was a significantly higher error rate per prescriber among the Novice Control group compared to the Experienced Control group (*P =* .026, 95% CI 0.031 to 0.397; f = 1.11; Table and Figure 3). However, there was no significant difference in error rate per prescriber between the Experimental Group and Experienced Control group.

There was also no significant difference in error rate per prescriber across the various grades of error severity type between the Experimental Group and Experienced Control group. However, there was a significantly higher minor error rate per prescriber among the Novice Control group compared to the Experienced Control group (*P =* .021, 95% CI 0.041 to 0.413, f = 1.10).

#### 3.3.3 patient‐level data

**FIGURE 4:**
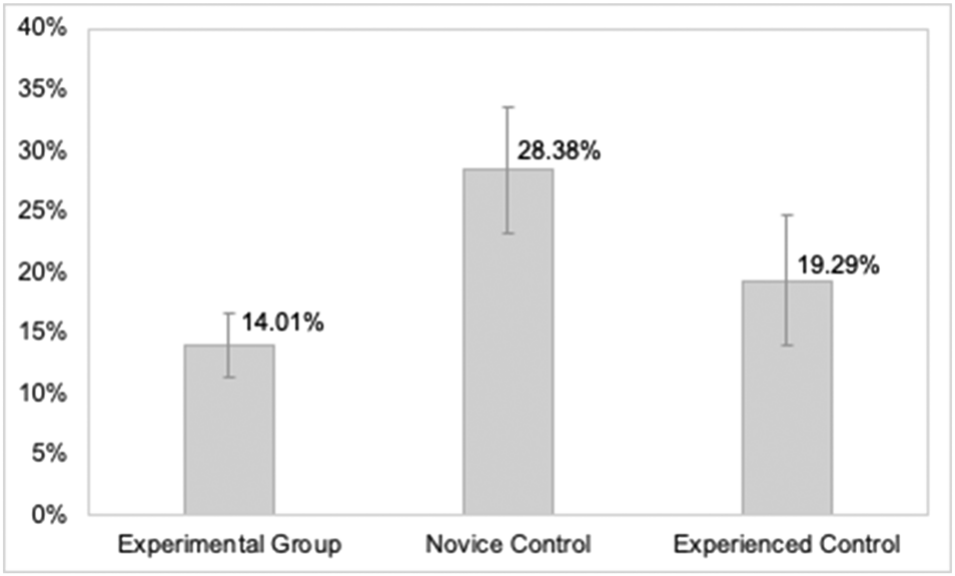
Error rates per patient (y‐axis) with confidence intervals by group of prescribers by whom the patients were seen/prescribed for Study 3: Department of Orthopaedics

Patients prescribed medicines by the Novice Control group had a significantly higher error rate per patient (28.38%) than patients prescribed medicines by the Experienced Control group (19.29%; *P =* .007, 95% CI 0.025 to 0.157 f = 0.12; Table and Figure 4). There was no significant difference in error rates for patients prescribed medicines by the Experimental Group and Experienced Control group. Patients prescribed medicines by the Experimental Group had the lowest error rate per patient compared with other groups (14.01%).

The same trend was also observed for minor error rates with patients seen by the Novice Control group subject to the most minor errors in comparison to patients seen by both other groups. Likewise, patients seen by the Experimental Group were subject to the fewest minor errors made in comparison to the patients seen by both other groups. Conversely, patients seen by the Experimental Group had a significantly lower rate of significant errors per patient (2.43%) compared to patients seen by the Experienced Control group (*P =* .04, 95% CI −0.068 to −0.001; f = 0.12). There was no significant difference in error rates among patients seen by the Novice Control group and Experienced Control group.

## 4 DISCUSSION

This research demonstrated that simulated clinical encounters and personalised, structured, video‐enhanced feedback using a deliberate practice approach is an effective intervention for improving the prescribing behaviour of Foundation Year doctors on four‐month training rotations across two NHS Trusts in the UK. The intervention was effective at reducing potential avoidable harm to patients, given that patients seen by the novice prescribers participating in the intervention experienced lower prescribing errors rates. This research addressed some of the concerns and gaps previously identified related to prescribing interventions^20^, ^21^, ^22^, ^23^, ^24^, ^54^ by demonstrating the impact of the intervention for improving clinical outcomes,^55^ namely, reducing prescribing errors rates, as well as confirming the transferability of the intervention across different clinical sub‐specialty contexts. Finally, the research suggested the intervention was cost‐effective for reducing the potential harm caused from prescribing errors thereby going some way toward attaining the WHO *Medication Without Harm* goal in the UK context.

Previous research has consistently identified that all prescribers make errors irrespective of experience, grade or professional background, but Foundation Year doctors make more compared to other prescriber groups.^6^, ^12^ The findings in this research also confirmed the same observation. This is the first study, however, to propose and demonstrate an effective intervention for improving the prescribing behaviour of novice prescribers to the level of experienced prescribers. In addition, this research suggests educational interventions (in our case personalised, structured and video‐enhanced feedback) underpinned by a deliberate practice approach are effective in changing and sustaining prescribing behaviours over at least a four‐month period in comparison to education that merely seeks to provide ‘a training experience' or promote a change in knowledge alone.

The three‐study research design was specifically constructed to address concerns raised in educational, training and patient safety research in relation to a lack of evidence for interventions in real‐world settings.^55^, ^56^ This research demonstrates that complex interventions designed to improve medical education, patient safety and practice can be operationalised in naturalistic healthcare settings without significant difficulty, whilst adopting a robust, experimental study design. That said, the findings from this research need to be investigated in other clinical contexts using a multicentre randomised study design to critically investigate the reproducibility of the intervention.

The impact of feedback on performance is widely reported across many domains and disciplines.^30^ However, the findings from this research raise a number of interesting issues about feedback given to Foundation Year doctors ranging from, first, practical issues related to method and timing, through to, second, philosophical considerations related to educational approach and ethics. First, Foundation Year doctors do not currently consistently receive personalised, structured feedback following simulation activities organised as part of their postgraduate training. This research demonstrated that the absence of such feedback may, at a minimum, have no effect, or, in extreme cases, could lead to worsening future prescribing behaviour in practice and a risk of patient harm. Second, factors such as low self‐confidence, inexperience in practice and fear of not appearing knowledgeable, may also affect the effectiveness of feedback among learners.^57^ This should be taken into account when delivering this or similar interventions in the future.

Previous research on prescribing interventions incorporating feedback do not demonstrate a consistent impact on outcomes.^29^, ^58^ This suggests that medical educators (or anyone delivering prescribing interventions) should not assume interventions delivered together with the provision of general feedback are enough to improve prescribing behaviour among junior doctors. The effect of video in facilitating feedback and deliberate practice,^59^ however, has been found to be particularly important for learning gains in a variety of contexts.^30^ In healthcare, two studies have successfully demonstrated the adoption of video‐based feedback; first, to improve surgical technical skills^60^ and, second, to reduce prescribing errors.^46^ These two interventions involved filming practice in situ. Whilst our research designed simulated clinical encounters with *real* patients, there remains a need for evaluating the feasibility of implementing interventions that go beyond technical skills on a greater scale.

The findings from this research have implications for the delivery of simulated encounters for Foundation Training and also simulations delivered as part of undergraduate medical curricula. Currently this type of training has greater emphasis on the *overlearning* of technical or psychomotor skills, such as advanced life support, rather than facilitating complex cognitive skills such as prescribing, which involves complicated problem‐solving and diagnostic decision‐making. Whilst training and assessment of competence in these skills are important among Foundation Year doctors, more simulation time should be given to improving *everyday skills* such as prescribing, safe handover,^61^ acting on results^62^ and undertaking ward rounds.^63^

The observed error rates calculated from individual's daily prescribing data, collected in Study 2 and 3, are notably greater than the group‐level error rates reported in the most cited previous study.^6^ There are four possible reasons attributed to this that need to be considered when comparing these results with other research. First, error rate data in this research is based on individual's prescribing activity, whilst previous research^6^ reports group‐level prescribing activity. Second, the underpinning methodology for collecting the baseline prescribing data was distinctive in our research. Data were collected continuously over a four‐month period across our studies, whereas a sampling day approach is a more conventional method of data collection.^6^ Third, the sampling approach taken to collect data in the most cited previous study^6^ was conducted across 19 acute hospital trusts in the North‐West region of England but data for individual trusts were not reported. The research reported in this manuscript reports on specific sites in the Midlands region of England; geographical variation across study sites may explain some error rates. Finally, differences in error severity were observed across the clinical sub‐specialities reported in this research, and only in Nephrology and Renal Transplantation were all four categories of error severity found. Furthermore, nephrology is reported to be the most complex prescribing clinical sub‐specialty.^64^ Other research^6^ does not report across sub‐specialty reducing the potential for learning.

This study demonstrated the intervention could be considered cost‐effective as outlined by the break‐even analysis reported in more detail in Appendix C. Making sense of cost‐effectiveness in a healthcare professions education context is complicated.^65^, ^66^ With respect to this study, the analysis was approached by comparing the cost of the intervention to an average cost for a medication error in the first study. Clearly the costs and benefits will vary across different sub‐specialty contexts.

## 5 LIMITATIONS

Given the voluntary nature of participation for this research from a sample population of Foundation Year doctors, sample sizes in this study were limited. As a consequence, randomising participants to conditions was not possible; consideration was also given to participation whilst ensuring clinical cover. Similarly, measuring impact on practice across subsequent sub‐specialty rotations, longitudinally, was not feasible. Foundation Year doctors rotate across hospitals and care settings within a region making consistent data collection not possible.

## 6 FURTHER RESEARCH

Future research should adopt a granular data collection method similar to the approach used in this research. The granular data collection allowed sub‐specialty level, error rate and error severity observations. This research provides a template for how such an intervention and an associated study may be designed and implemented in the NHS. If this intervention is adopted, research should create long‐term changes in participant learning, practice and patient safety behaviours. Finally, this research involved study sites with written inpatient prescription charts, therefore the effectiveness of this intervention using electronic prescribing systems requires further investigation.^67^

## 7 CONCLUSION

Simulated clinical encounters with personalised, structured and video‐enhanced feedback using a deliberate practice approach significantly improves the prescribing performance and patient safety behaviours of Foundation Year doctors. This intervention was demonstrated to be effective across three different clinical sub‐specialties in medicine (nephrology) and surgery (general and orthopaedics). The intervention is cost effective and has the potential to reduce the avoidable harm resulting from poor prescribing. The intervention (simulated clinical encounters with personalised, structured and video‐enhanced feedback using a deliberate practice approach) is an important contribution to the WHO's Global Patient Safety challenge, *Medication Without Harm.*

## COMPETING INTERESTS

There are no competing interests to declare.

## CONTRIBUTORS

W.G. and R.P. were involved in the conception of the study and obtaining funding. W.G., M.W.S., M.M.M., A.B., A.N., R.J., J.S. and R.P. contributed to the intervention design. W.G., M.W.S., S.W., M.M.M., Z.W., J.S. and R.P. contributed to the study design including data collection and analysis. All authors contributed to drafting and revising the manuscript.

## Supporting information


**DATA S1** **TABLE C1** Cost of intervention
**TABLE C2** Costs of adverse drug events
**TABLE C3** Assigned error severity and associated probability
**TABLE C4** Adjusted cost of errors
**TABLE C5** Break‐even number of errors to cover intervention costs
**TABLE C6** Reduction in errors by error severity

**DATA S2** Supporting information

**DATA S3** Supporting information

## Data Availability

The data that support the findings of this study are available on request from the corresponding author. The data are not publicly available due to privacy or ethical restrictions.
