# Supplementary figures and images for: Improving junior doctor medicine prescribing and patient safety: An intervention using personalised, structured, video‐enhanced feedback and deliberate practice
Source: Br J Clin Pharmacol. 2020 May 18;86(11):2234–46. doi: 10.1111/bcp.14325 (PMC7576627; doi:10.1111/bcp.14325)

**Appendix B**

**Prescribing error data collection form**

**
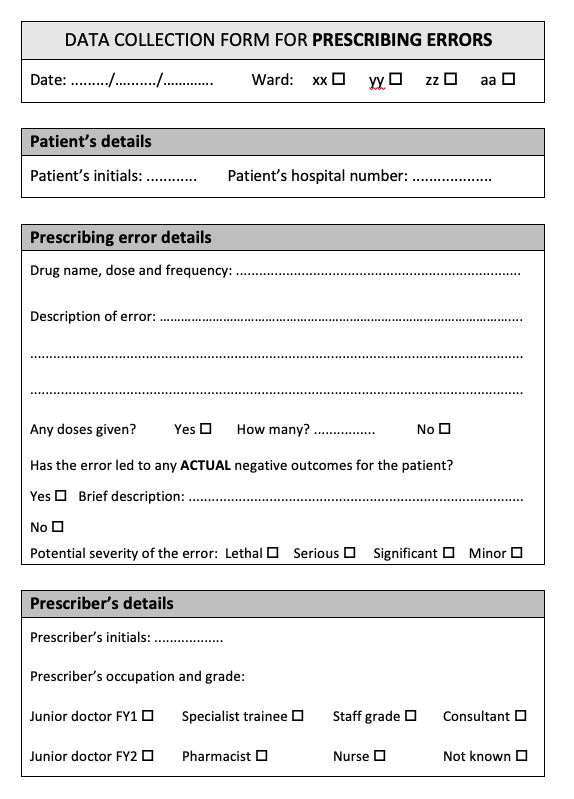
**

Supplement: Supplementary file 1 — DATA S1 TABLE C1 Cost of intervention TABLE C2 Costs of adverse drug events TABLE C3 Assigned error severity and associated probability TABLE C4 Adjusted cost of errors TABLE C5 Break‐even number of errors to cover intervention costs TABLE C6 Reduction in errors by error severity [file BCP-86-2234-s001.docx]
