# Supplementary material for: Improving junior doctor medicine prescribing and patient safety: An intervention using personalised, structured, video‐enhanced feedback and deliberate practice
Source: Br J Clin Pharmacol. 2020 May 18;86(11):2234–46. doi: 10.1111/bcp.14325 (PMC7576627; doi:10.1111/bcp.14325)
Supplement: Supplementary file 2 — DATA S2 Supporting information [file BCP-86-2234-s002.docx]

Figure C1: Visual representation of the break-even analysis with the cost of the intervention in orange
