# Supplementary material for: Improving junior doctor medicine prescribing and patient safety: An intervention using personalised, structured, video‐enhanced feedback and deliberate practice
Source: Br J Clin Pharmacol. 2020 May 18;86(11):2234–46. doi: 10.1111/bcp.14325 (PMC7576627; doi:10.1111/bcp.14325)
Supplement: Supplementary file 3 — DATA S3 Supporting information [file BCP-86-2234-s003.docx]

**APPENDICES**

**Appendix A**

**The intervention**:

At the beginning of this research, prior to Study 1, a participatory design workshop was held to inform the design of the intervention. Participants included a patient, a junior doctor (author xx), a medical educator (author x), a human factors specialist (author y), and a pharmacist (author Z). The outcome of the workshop was refined to ensure that the intervention was feasible and repeatable.

The intervention is made up of two parts: simulations; and personalised, structured and video-enhanced feedback.

**Simulations**:

Simulations are designed for junior doctors’ participation to last 120 minutes:

0-15mins: Introduction

16-95mins: Four 20-minute patient–doctor scenarios, which require the doctors to:

- - 1. Familiarise themselves with the patient, e.g. take history and read handover notes,
    2. Diagnose,
    3. Prescribe.

96-115mins: Write handover notes.

116-120mins: Lead board round with senior clinician.

The small ward-round incorporated a handover followed by a board-round designed to recognise the act of prescribing, as a technical task, is a small part of the prescribing process. Four patients are considered appropriate and feasible. Each participating doctor receives a handover report about each of the four patients from the nurses. Thereafter, they review each patient, during which they are required to take a medical history and conduct a physical examination to develop a list of differential diagnoses or clinical problems. Alongside the documentation of these activities, the doctors construct a patient management plan and adjust drug or fluid charts as appropriate. Doctors complete each consultation by explaining their management plan to the patient and negotiating a shared outcome. At the end of the activity, doctors are required to update the original handover list and communicate the patient management plan in a board-round to a senior doctor.

**Personalised, structured and video-enhanced feedback**:

Feedback is scheduled with the individual junior doctor to fit with their rota and to prevent adverse outcomes on patient safety which would occur if they were removed when scheduled to be clinically facing. Doctors are free to organise one or more sessions depending on their availability. During feedback, the video recordings are reviewed, and structured feedback is delivered by a clinical educator. Videos of the patient–doctor interactions are viewed with pauses to allow reflection on the doctor’s interaction with the patient. These reflections on the approach then inform specific areas of improvement to be addressed by junior doctors in their deliberate practice. Video feedback is as close to the simulations as possible.

**Local trust team**:

A local Trust team is created to design and run the simulations. This included in our cases: patient advocate, executive member of the Trust to act as advocate for the intervention, a rota manager to ensure junior doctors would be available, senior clinician, nursing staff and pharmacists.

**Clinical encounter scenarios**:

Patient scenarios are written by the local Trust team practice members, i.e. medic, pharmacist and nurse. These are based on Trust priorities for the participating medical specialty and account for the medical history of the participating patients. Clinical encounter scenarios are written around each participating patient’s own clinical history and designed around the participating NHS Trust priorities and guidelines; for example, by building on recent serious untoward incidents (SUIs). Patients who are not actors then mimic real symptoms. Simulations should be taxing but are designed to be feasible for a post-graduate junior doctor to achieve. Twenty minutes is considered a reasonable length of time for the simulations and complexity of scenario. Props are designed for the scenarios to be realistic, e.g. authentic- looking general practitioner letters, computers on wheels available to look at scans, and prescribing charts with pre-existing entries.

**Patients**:

In our case, four patients agreed to participate in each study; these were outpatients. Patients should be briefed on the scenarios and given example answers to likely medical questions.

**Patient rooms**:

Patients are based in their own bay or in their own room. This is required to ensure that video and audio is of sufficient quality to enable feedback.

**Briefing room**:

Doctors are given a briefing on the simulations process prior to embarking on the simulations.

**Authenticity**:

Simulations must be purposively designed not to be associated with a test or prior simulated assessment. Participants should be told that the simulated environment is being used to support the development of the individual participant and not as a formal evaluation of practice.

To recreate the interacting factors associated with medical and prescribing errors, and a busy, real working environment, nurses and pharmacists should be available to be asked for advice by the participating doctors if required. Nurses can also regularly interrupt doctors and interact with patients to take observations and check their current state.

Nurses, pharmacists and senior doctors are available for consultation. Patients, props, patient–doctor encounters and the clinical environment are purposively designed to feel authentic. For example, props for scenarios are available, including authentic-looking stools and commonplace objects that can cause complex medication interaction, such as orange juice. In addition, authentic patient scans are available as is normal, on COWs (Computers on Wheels) and letters from GPs are designed to look authentic.

< APPENDIX B HERE >

**Appendix C**

**Break-even analysis**

Costing adverse drug events (ADEs) is debated and complex.[64, 65] This supplement provides further information on the costs of the intervention, the resources used for the intervention, and a break-even analysis of cost. This was chosen as a simple approach to understanding the costs of ADEs.

**Outcome measures**

For the break-even analysis, the main outcome measure used is the reduction in written prescription errors.

**Resources used and costs**

All costs obtained for this analysis are from the perspective of the UK NHS and relate to the direct costs of undertaking the intervention. Table C1 summarises the direct resources used for the intervention and the associated costs.

< TABLE C1 NEAR HERE >

**Cost of adverse drug events**

In order to ascertain the costs associated with an ADE, a micro costing analysis was undertaken of 10 cases in the Renal unit. In all cases, the costs associated with the event have been calculated from the point the patient was admitted to the Renal unit. In order to derive a total cost for a given ADE, the direct costs resulting from increased bed days and extra interventions were calculated, as well as other core costs such as additional medication or any admissions onto high-dependency facilities. A total direct cost for each case was derived thereafter by the research team. Table C2 provides a summary of the costs of ADEs for all 10 cases.

< TABLE C2 NEAR HERE >

The average cost of an ADE in the Renal unit based on this estimate is therefore £14,586. It is assumed that the cost of ADEs calculated in this instance correspond to potentially lethal ADEs to maintain a conservative bias. In addition, we have also taken into consideration the probability of ADE occurrence. This classification is broadly based on previous studies that have calculated the probabilities of ADEs.[68] Table C3 provides the probabilities associated with each severity classification of the error. It is also assumed that had these prescription errors not been detected by a pharmacist, they would all likely lead to an ADE.

< TABLE C3 NEAR HERE >

**Break-even analysis**

The break-even analysis will provide an estimate of how many prescription errors need to be reduced in order to achieve cost savings equivalent to the cost of intervention. The following formula has therefore been used:

*Break-even number of errors = Cost of intervention/probability adjusted cost ADE*

To reiterate, the average cost of an ADE as per our micro-costing analysis in the Renal unit is £14,586. The full average cost of an ADE has been assigned to the severity category of “lethal”.

Based on this, and the associated probabilities, the break-even analysis would suggest that within each category, the following number of errors will need to be reduced to cover intervention costs (Table C4 and Table C5).

< TABLE C4 NEAR HERE >

< TABLE C5 NEAR HERE >

The cost of the intervention, £22,270, can be covered by reducing 16 minor errors, 4 significant errors, 3 serious errors, or 2 lethal errors on their own.

The reduction in errors as a result of the EPIFFANY intervention is summarised in Table C6. This is represented in Figure C1.

< FIGURE C1 HERE >

Table C1: Cost of intervention*

| Resource | Requirement | Notes | Intervention cost |
| --- | --- | --- | --- |
| Videoed simulation training | Simulation centre days including staff, nurses etc. per day | Includes 4 bay hires, bench top rooms, day ward seminar room, handover bay, 3 clinical skills facilitators, technician and consumables (4 days) | £8,300 |
|  | Recording material (camera etc.) per day | One camera required for each bay, hence in total 4 cameras. Assuming cost of hiring at £200 per camera per day (4 days) | £3,200 |
|  | Admin. support for coordination for the 4 days | Assuming agenda for pay scale Band 2 | £240 |
|  | Consultant for board round 3 hours per simulation day x 4 days | Assuming board round lasts an hour a session, two sessions a day, 4 simulation days. Assuming basic pay scale of £101,451 based on Agenda for Change | £576 |
|  | Patients actors x 4 per simulation x 4 days | Assuming a rate of £15 an hour, 8 hours per day | £1,920 |
|  | CT doctor x 4 days | Assuming Agenda for Scale Band 6 | £400 |
|  | SpR** for 4 days | Assuming year 7 SpR pay scale | £1,000 |
| Feedback session with supervisor | 3 hours x 4 sessions | 8 doctors per session, 32 doctors total. Assuming year 7 SpR pay scale (4 sessions in total) | £375 |
| Pharmacist | Teaching/feedback sessions 1 hour per day | 21 sessions in total (3 working days) | £645 |
| Group debrief | Assigned SpR supervisor 1 day | Assuming year 7 SpR pay scale | £250 |
| Project management | Band 6 Agenda for Change pay scale – 0.6 WTE*** | Coordination of the project, meetings and sessions, for 4 months | £5,364 |
| Total cost of intervention | | | **£22,270** |

*Costs calculated for year 2014/15; ** SpR = Specialist Registrar; *** WTE = whole time equivalent.

Table C2: Costs of adverse drug events

| Patient number | Bed costs £ | Intervention  costs £ | Medication  costs £ | High depend- ency bed day costs £ | Total  costs £ | Days stay |
| --- | --- | --- | --- | --- | --- | --- |
| 1 | 8,050.00 | 4,331.00 | 114.28 | 16,812.00 | 29,307.28 | 23 |
| 2 | 15,400.00 | 7,775.00 | 35.30 |  | 23,210.30 | 44 |
| 3 | 700.00 | 188.00 | 7.42 |  | 895.42 | 2 |
| 4 | 4,200.00 | 1,205.00 | 101.94 |  | 5,506.94 | 12 |
| 5 | 2,450.00 | 1,152.00 | 21.26 | 1,401.00 | 5,024.26 | 7 |
| 6 | 5,600.00 | 1,270.00 | 176.49 |  | 7,046.49 | 16 |
| 7 | 5,600.00 | 2,727.00 | 73.87 |  | 8,400.87 | 16 |
| 8 | 7,000.00 | 3,949.00 | 89.45 | 1,401.00 | 12,439.45 | 20 |
| 9 | 8,400.00 | 3,151.00 | 1,329.90 |  | 12,880.90 | 24 |
| 10 | 23,450.00 | 12,657.00 | 836.90 | 4,203.00 | 41,146.90 | 61 |
| Total | 80,850.00 | 38,405.00 | 2,786.81 | 23,817.00 | 145,858.81 | 225 |

Table C3: Assigned error severity and associated probability

| Error severity | Associated probability of harm |
| --- | --- |
| Minor | 0.1 |
| Significant | 0.4 |
| Serious | 0.6 |
| Lethal | 0.9 |

Table C4: Adjusted cost of errors

|  | Minor £ | Significant £ | Serious £ | Lethal £ |
| --- | --- | --- | --- | --- |
| Average cost of ADE | 14,586 | 14,586 | 14,586 | 14,586 |
| Probability of ADE | 0.1 | 0.4 | 0.6 | 0.9 |
| Adjusted cost | 1,458.6 | 5,834.4 | 8,751.6 | 13,127.4 |

Table C5: Break-even number of errors to cover intervention costs

|  | Minor £ | Significant £ | Serious £ | Lethal £ |
| --- | --- | --- | --- | --- |
| Adjusted cost | 1,458.6 | 5,834.4 | 8,751.6 | 13,127.4 |
| Intervention cost | 22,270 | 22,270 | 22,270 | 22,270 |
| Break-even (intervention cost/adjusted cost) | 15.26807 | 3.817016 | 2.544678 | 1.696452 |

Table C6: Reduction in errors by error severity

|  | Number of errors | | |
| --- | --- | --- | --- |
| Error severity | Experimental Group 1  (without personalised, structured, video-enhanced feedback) | Experimental Group 2 (with personalised, structured, video-enhanced feedback) | Reduction |
| Minor | 41 | 32 | 9 |
| Significant | 52 | 34 | 18 |
| Serious | 9 | 6 | 3 |
| Lethal | 0 | 0 | 0 |
| Total | 102 | 72 | 30 |
